# Intraoperative Fluorescence-Guided Resection of High-Grade Malignant Gliomas Using 5-Aminolevulinic Acid–Induced Porphyrins: A Systematic Review and Meta-Analysis of Prospective Studies

**DOI:** 10.1371/journal.pone.0063682

**Published:** 2013-05-28

**Authors:** Shiguang Zhao, Jianing Wu, Chunlei Wang, Huailei Liu, Xingli Dong, Chen Shi, Changbin Shi, Yaohua Liu, Lei Teng, Dayong Han, Xiaofeng Chen, Guang Yang, Ligang Wang, Chen Shen, Huadong Li

**Affiliations:** 1 Department of Neurosurgery, The First Affiliated Hospital of Harbin Medical University, Harbin, China; 2 Brain Science Institute of Harbin Medical University, Harbin, China; 3 Department of Biochemistry and Molecular Biology, Harbin Medical University, Harbin, China; 4 Department of Neurosurgery, New York University Langone Medical Center and School of Medicine, New York, New York, United States of America; 5 Section of Neurosurgery, Department of Surgery, The University of Chicago Medical Center and Pritzker School of Medicine, Chicago, Illinois, United States of America; Glasgow University, United Kingdom

## Abstract

**Background:**

We performed a systematic review and meta-analysis to address the (added) value of intraoperative 5-aminolevulinic acid (5-ALA)-guided resection of high-grade malignant gliomas compared with conventional neuronavigation-guided resection, with respect to diagnostic accuracy, extent of tumor resection, safety, and survival.

**Methods and Findings:**

An electronic database search of Medline, Embase, and the Cochrane Library was undertaken. The review process followed the guidelines of the Cochrane Collaboration. 10 studies matched all selection criteria, and were thus used for qualitative synthesis. 5-ALA-guided resection demonstrated an overall sensitivity of 0.87 (95% confidence interval [CI], 0.81–0.92), specificity of 0.89 (95% CI, 0.79–0.94), positive likelihood ratio (LR) of 7.62 (95% CI, 3.87–15.01), negative LR of 0.14 (95% CI, 0.09–0.23), and diagnostic odds ratio (OR) of 53.06 (95% CI, 18.70–150.51). Summary receiver operating characteristic curves (SROC) showed an area under curve (AUC) of 94%. Contrast-enhancing tumor was completely resected in patients assigned 5-ALA as compared with patients assigned white light. Patients in the 5-ALA group had higher 6-month progression free survival and overall survival than those in the white light group.

**Conclusion:**

Based on available literature, there is level 2 evidence that 5-ALA-guided surgery is more effective than conventional neuronavigation-guided surgery in increasing diagnostic accuracy and extent of tumor resection, enhancing quality of life, or prolonging survival in patients with high-grade malignant gliomas.

## Introduction

Malignant gliomas are locally invasive brain tumors which inevitably lead to death within two years after diagnosis [1], [2]. The first reported case of glioma resection was performed by Rickman Godlee in 1884 [3]. As far as surgery is concerned, resection of the main tumor mass to achieve debulking in symptomatic patients is a widely accepted strategy [4]. Extent of tumor resection (EOTR) is increasingly accepted as critical to optimal surgical treatment and patient outcome [1], [5]. Since malignant gliomas do not have a distinct margin between the tumor mass and the surrounding brain, achieving gross total tumor resection represents a major challenge to the neurosurgeon. Numerous surgical technologies have been developed to facilitate optimal resection, many of which function to guide the surgeon during resection. These technologies include intraoperative magnetic resonance imaging (MRI), intraoperative ultra-sound, intraoperative computed tomography (CT), and fluorescence-guided surgery with 5-aminolevulinic acid (5-ALA).

The implementation of 5-ALA-guided surgery is considered to be much simpler and less costly than other types of surgery for patients with malignant glioma [6]. 5-ALA is a precursor in the hemoglobin synthesis pathway which elicits the synthesis and accumulation of fluorescent porphyrins in various epithelia and cancerous tissues [7]. Oral administration of 5-ALA several hours before surgery leads to the preferential accumulation of protoporphyrin IX (PpIX) within glioma cells. Under blue-violet light conditions, the PpIX emits light in the red region of the visible spectrum, enabling identification of tumor tissue that would otherwise be difficult to distinguish from normal brain [8]–[10]. A large, multicentre phase III randomised control trial comparing 5-ALA-induced fluorescence-guided surgery with conventional white light surgery for malignant glioma was a landmark in fluorescence-guided surgical resection [11], [12]. 5-ALA guided resection of primary malignant brain tumors was found to be beneficial, resulting in a significantly higher resection rate, which translated into a longer progression-free interval, as well as a longer median survival time.

Since the introduction of 5-ALA, many studies have reported on the (added) value of 5-ALA-guided surgery. However, to the best of our knowledge, no systematic review and meta-analysis on this topic has been published. 5-ALA has been approved for intracranial use in Europe, Canada, and Japan, but not in the United States [8]. Widespread use of 5-ALA should be based on evidence.

We did a systematic review and meta-analysis of the literature in accordance with the Preferred Reporting Items for Systematic Reviews and Meta-Analyses (PRISMA) statement [13]. Our objective was to address the (added) value of 5-ALA-guided resection of high-grade malignant gliomas particularly glioblastoma multiforme (GBM), compared with conventional neuronavigation-guided resection, with respect to diagnostic accuracy, EOTR, safety, and survival.

## Methods

### Research Protocol

Our research protocol consisted of the detailed research question, search strategy, screening criteria for titles, abstracts, and full-text articles. The detailed research question was formed by the patient, intervention, comparator, outcome, study design (PICOS) approach. Our objective was to address the (added) value of 5-ALA-induced fluorescence-guided resection of high-grade malignant gliomas particularly GBM compared with conventional neuronavigation-guided resection, with respect to diagnostic accuracy, EOTR, safety, and survival.

We considered only the two most reliable types of studies. These were prospective studies on diagnostic accuracy and therapeutic outcome. Only trials designed for level 1 and 2 were included (Level 1: High quality randomized trial or prospective study; Level 2: Lesser quality RCT; prospective comparative study). Diagnostic accuracy was investigated by examining tissue biopsy samples surgically excised by 5-ALA-induced fluorescence-guided surgery in combination with conventional neuronavigation-guided surgery. Patients had to have a suspected or proven high-grade malignant gliomas. EOTR, safety, and survival were assessed after randomising patients under-going resection with either white light (WL) alone or combining with 5-ALA. Both procedures were only considered in primary gliomas. A lesion-based measure was preferred over a patient-based one for clinical relevance.

Search databases were Medline (using PubMed), Embase (using Ovid), and the Cochrane Library. Search queries were optimised for each specific database.

Titles and abstracts were screened and included if they represented randomised or cohort studies of patients with high-grade malignant gliomas who received neurosurgical intervention using 5-ALA. Duplicate records were deleted. Studies that specifically reported on a pediatric population or focused on chemotherapy or radiosurgery, radiotherapy were excluded. Studies in abstract form, case reports, and a language other than English were excluded. Studies including fewer than 20 patients or 100 biopsies were not retained. Of the remaining records, full-text articles were assessed according to the same criteria, with one additional exclusion criterion (overlapping data). Shiguang Zhao and Jianing Wu set up the research protocol, and searches were done independently by Huailei Liu and Chunlei Wang. Xingli Dong served as an independent third reviewer in cases where opinion differed between the two reviewers.

### Eligibility Criteria and Search Strategy

The PICOS research question was a foundation for study selection. An electronic database search of Medline, Embase, and the Cochrane Library was undertaken. Since 5-ALA is a somewhat new technique, the topic itself limited the publication dates (last search was done Oct. 15th, 2012). We used PubMed as our primary data source, and searched the above databases using the following MeSH terms and search strategies:

Medline: #1 Brain neoplasms; #2 Glioma; #3 Glioblastoma; #4 (#1 OR #2 OR #3); #5 Photosensitizing agents/diagnostic use*; #6 Protoporphyrins/diagnostic use; #7 Photosensitizing agents; #8 Protoporphyrins; #9 Aminolevulinic acid; #10 Protoporphyrin IX; #11 (#5 OR #6 OR #7 OR #8 OR #9 OR #10); #12 (#4 AND #11).

The Ovid query with thesaurus terms was: # 1 (Protoporphyrins or aminolevulinic acid or protoporphyrin IX).mp. [mp = tx, bt, ti, ab, ct]; # 2 (Brain neoplasms or glioma or glioblastoma).mp. [mp = tx, bt, ti, ab, ct]; # 3 (# 1 and # 2).

The Cochrane Library query with keywords was: #1 (MeSH descriptor Brain neoplasms explode all trees); #2 (glioma):kw or (glioblastoma):kw; #3 (#1 OR #2); #4 (Protoporphyrins):kw or (Aminolevulinic acid):kw or (protoporphyrin IX):kw; #5 (#3 AND #4).

### Data Extraction and Quality Assessment

After screening the database search results, full-text assessment was done for study selection. The review process was performed according to the guidelines of the Cochrane Collaboration. All data were extracted into a computer-based spread-sheet. The authors, publication year, study type, and clinical data were included. In addition, the following clinical data were extracted for each diagnostic accuracy trial, where available: specificity, sensitivity, true-positive (TP), false-positive (FP), true-negative (TN), and false-negative (FN). If such information was not reported specifically in the original article, we performed an analysis with the reported data to generate desired values when possible. Diagnostic accuracy was assessed by sensitivity, specificity, positive likelihood ratio (LR), negative LR, and diagnostic odds ratio (OR). EOTR was assessed by comparing residual contrast-enhancing tumor volume. Safety was assessed by comparing adverse events (AEs), serious adverse events (SAEs), the NIH-SS score, and the KPS score. Survival was assessed by progression-free survival and overall survival time. The quality and applicability of studies were assessed using checklists of the Quality Assessment of Diagnostic Accuracy Studies (QUADAS, scale 0–14). QUADAS is an evidence based tool to be used for the quality assessment of diagnostic accuracy studies in systematic reviews. It consists of 14 items phrased as questions, each of which should be scored a “yes”, “no” or “unclear” that examine bias in the study [14].

### Statistical Analysis

All analyses were performed at the biopsy level. All samples were examined for histologic diagnosis according to the World Health Organization (WHO) classification of tumors of the central nervous system [15]. Primary data synthesis was performed within the bivariate mixed-effects binary regression modeling framework with Stata 11 (StataCorp). The bivariate fixed-effects regression model was employed for specificity analysis, and random-effects regression model for sensitivity analysis. In the fixed-effects regression model, the results of individual studies were pooled using weights that depended on the sample size of the study, whereas in the random-effects regression model, each study was weighted equally [16]. Average sensitivity, specificity, LR, OR, and 95% confidence interval (CI) were calculated from the maximum likelihood estimates, and graphically assessed by the summary receiver operating characteristic curves (SROC). Heterogeneity of the results between studies was assessed graphically on Forest plots and statistically using the *x* ^2^ test and Cochran Q. I-squared (*I^2^*) described the percentage of total variation across studies due to heterogeneity rather than chance, and was also used as a measure to quantify the amount of heterogeneity. *I^2^*>50% suggested heterogeneity [17]. Publication bias was assessed by a Funnel plot asymmetry test [18]. Statistics were performed with Stata 11 (StataCorp), and a *P* value less than 0.05 was considered statistically significant.

## Results

### Identification and Characteristics of Studies

**Figure 1:**
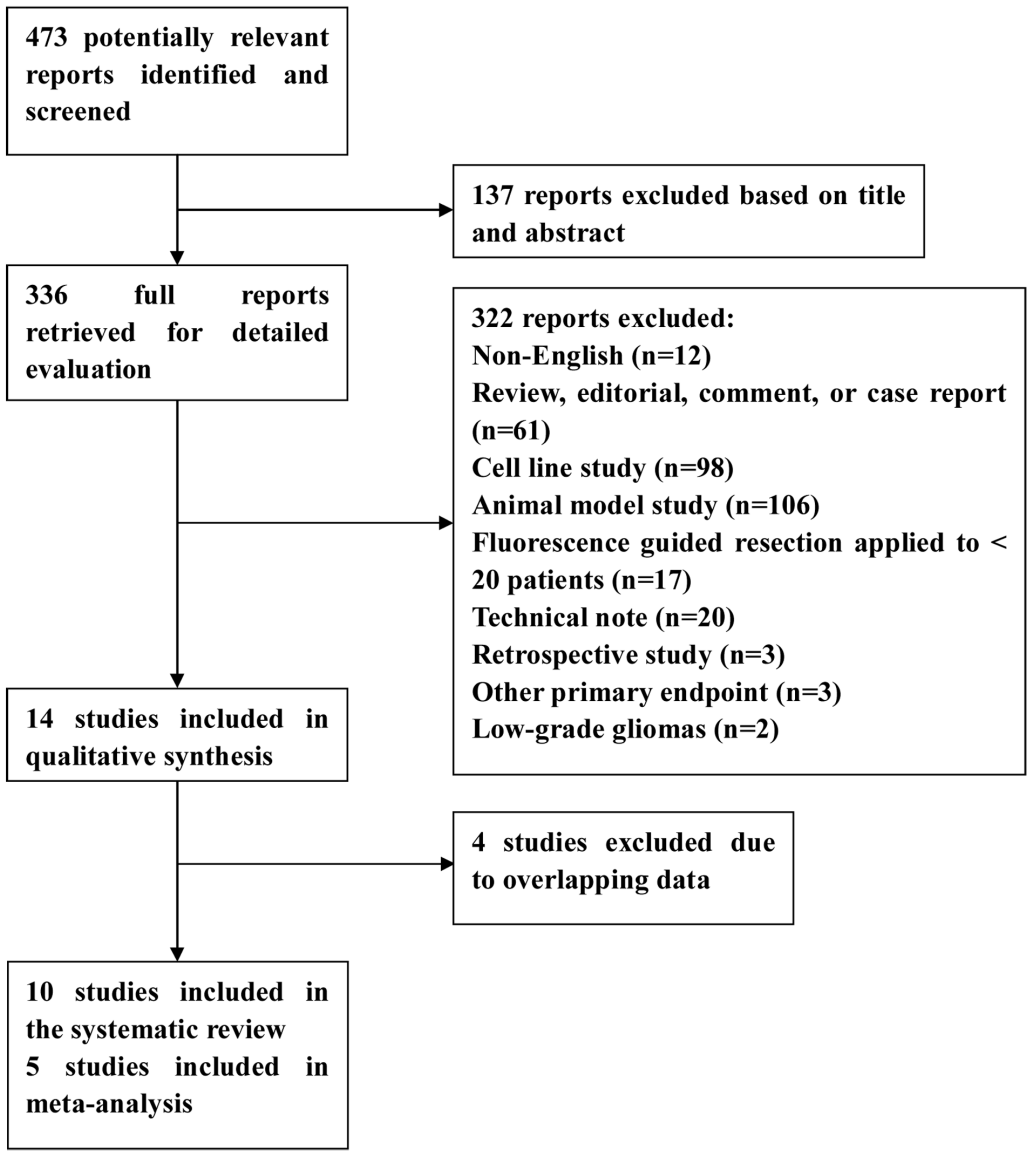
Flow diagram outlining the study selection process.

**Figure 2:**
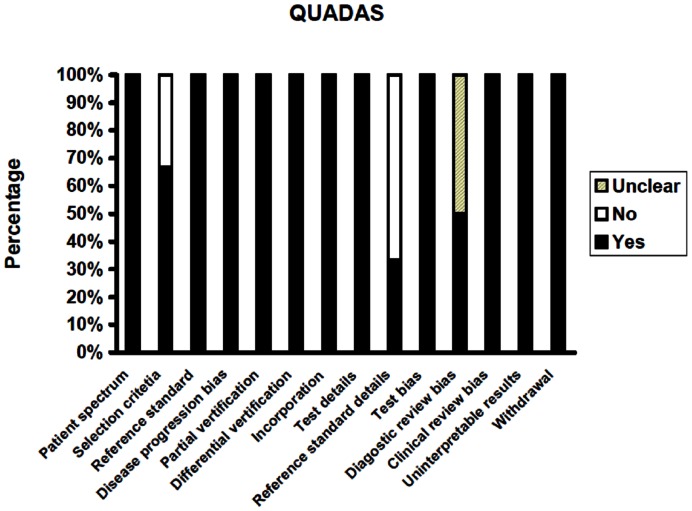
Evaluation of quality of included studies using the QUADAS tool.

Our search identified 473 potentially relevant reports, of which 336 were retrieved for detailed evaluation (Figure 1). Totally, 322 reports including some clinical trials were excluded for the reasons given in Figure 1 and Table 1. A total of 14 study reports met the inclusion criteria, including 4 reports from the same study. The remaining 10 articles were used for quantitative synthesis [4], [11], [12], [19]–[21], [23]–[26], including seven prospective and three randomised controlled trials (RCT). Studies where the prospective or retrospective design was not specified in the full-text article were assessed independently by both reviewers, who agreed in all cases. Publication years were between 2000 and 2012. Most studies gave inclusion and exclusion criteria for patient selection. Six studies included data on diagnostic accuracy of GBM [4], [19], [21], [23], [24], [26]. Five of these articles published biopsy-based detection rates, and thus included in our meta-analysis [4], [19], [21], [23], [26]. Survival data were available in six of 10 studies [11], [12], [20], [23], [24], [26]. Four articles were published by the ALA-Glioma study group [11], [12], [20], [26]. Detailed study design parameters for the selected studies are shown in Table 2. Table 3 shows the data of studies on diagnostic accuracy of GBM. Results of distribution of study design characteristics in six diagnostic studies according to QUADAS items are shown in Figure 2.

**Table 1:** Exclusion of clinical trials and reasons for exclusion.

Publication	Reason for exclusion
Ewelt et al. [22]	Low-grade gliomas
Valdes et al. [27]	Primary endpoint is quantitative ex vivo tissue measurements of PpIX
Utsuki et al. [28]	Retrospective study
Tsugu et al. [29]	Retrospective study
Stummer et al. [30]	Primary endpoint is the influence of the degree of resection on survival
Schucht et al. [31]	Retrospective study
Nabavi et al. [32]	Recurrent malignant glioma study
Panciani et al. [33]	Overlapping data with Panciani et al. [19]
Idoate et al. [34]	Overlapping data with Diez Valle et al. [23]
Floeth et al. [35]	Low-grade gliomas
Pichlmeier et al. [36]	Overlapping data with Stummer et al. [9]
Stepp et al. [37]	Overlapping data with Stummer et al. [9]

**Table 2:** Study design parameters of studies included.

Publication	Study Type	Mean (range) age (years)	Inclusion criteria	Exclusion criteria	Recruitment Time	Primary endpoint	Measurement of primary endpoint
Panciani et al.(2012)	Prospectivestudy	(43–79)	GBM	Eloquent areas. More than onecontrast-enhancing lesion.	Nov.2008 to Mar.2010	Diagnostic accuracy	Neuropathology
Stummer et al.(2011)	RCT	61.0 (23–73)	WHO Grade III/IV glioma	Low-grade gliomas	NS	EOTR, safety, survival	CE on EPMRI, KPS,NIH-SS score, event-free survival rate
Stummer et al.(2011)	ProspectiveStudy	60.7 (21–80 )	WHO Grade III/IV glioma	Conditions prohibiting the administration of 5-ALA	Jan.10, 2005 to Nov. 26,2007.	Safety, and survival	Adverse event, KPS, overall survival rate
Roberts et al.(2011)	ProspectiveStudy	63.8 (52–80)	GBM	Conditions prohibiting the administration of 5-ALA	Aug. 2008 and Apr. 2009	Diagnostic accuracy	Neuropathology
Diez Valle et al.(2011)	ProspectiveStudy	58.4 (30–73)	GBM	Bilateral tumor, multiple distantlesions	Oct. 2007 to Jun. 2009	Diagnostic accuracy, EOTR, safety, survival	Neuropathology, CE on EPMRI, adverse event, PFS, overall survival
Eljamel et al. (2009)	ProspectiveStudy	NS	NS	NS	NS	Diagnostic accuracy	Neuropathology
Hefti et al. (2008)	ProspectiveStudy	NS	Malignant glioma	NS	May 2006 to May 2007	Diagnostic accuracy	Neuropathology
Eljamel et al. (2008)	RCT	59.6	GBM	Whose diagnosis was not GBM	NS	Safety, survival	Adverse event, KPS, estimated survival
Stummer et al.(2006)	RCT	60 (23–73)	WHO Grade III/IV glioma	Low-grade glioma	Oct. 11,1999 to Jul.19, 2004	EOTR, survival	CE on EPMRI, KPS, progression-free survival
Stummer et al.(2000)	ProspectiveStudy	53.8 (31–69)	GBM	Conditions prohibiting the administration of 5-ALA	Dec. 1995 to Dec. 1998	Diagnostic accuracy, EOTR, survival	Neuropathology, CE on EPMRI, time from surgery to death

Abbreviations: RCT, randomised control trial; NS, not specified; EOTR, extent of tumor resection; GBM, glioblastoma multiforme; KPS, Karnofsky performance score; CE, contrast enhancement; EPMRI, early postoperative MRI; PFS, progression-free survival.

**Table 3:** Prospective trials accepted for analysis.

Publication	TRUE positive	FALSE positive	FALSE negative	TRUE negative	Sensitivity (95% CI)	Specificity (95% CI)	Positive LR (95% CI)	Negative LR (95% CI)	Diagnostic OR (95% CI)
Panciani et al. (2012)	41	5	4	42	0.91(0.79–0.98)	0.89(0.77–0.96)	8.56(3.72–19.71)	0.10(0.04–0.25)	86.10(21.59–343.41)
Roberts et al. (2011)	82	4	28	10	0.75(0.65–0.82)	0.71(0.42–0.92)	2.61(1.13–6.02)	0.36(0.22–0.56)	7.32(2.13–25.21)
Diez Valle et al. (2011)	142	2	12	24	0.92(0.87–0.96)	0.92(0.75–0.99)	11.99(3.16–45.43)	0.08(0.05–0.15)	142.00(29.89–674.53)
Hefti et al. (2008)	73	3	11	17	0.87(0.78–0.93)	0.85(0.62–0.97)	5.79(2.03–16.50)	0.15(0.09–0.28)	37.61(9.45–149.69)
Stummer et al. (2000)	211	1	26	26	0.89(0.84–0.93)	0.96(0.81–1.00)	24.04(3.51–164.59)	0.11(0.08–0.16)	211.00(27.48–1620.16)
Combined					0.87(0.81–0.92)	0.89(0.79–0.94)	7.62(3.87–15.01)	0.14(0.09–0.23)	53.06(18.70–150.51)

### Diagnostic Accuracy

Six studies [4], [19], [21], [23], [24], [26] reported the data on diagnostic accuracy of GBM with 5-ALA, five of which were lesion-based [4], [19], [21], [23], [26]. The patient-based study did not report data that could be used to construct or calculate TP, FP, TN and FN results [24]. Five articles were selected for data extraction and data analysis. Seven hundred and sixty-four brain lesions were included from the five studies.

**Figure 3:**
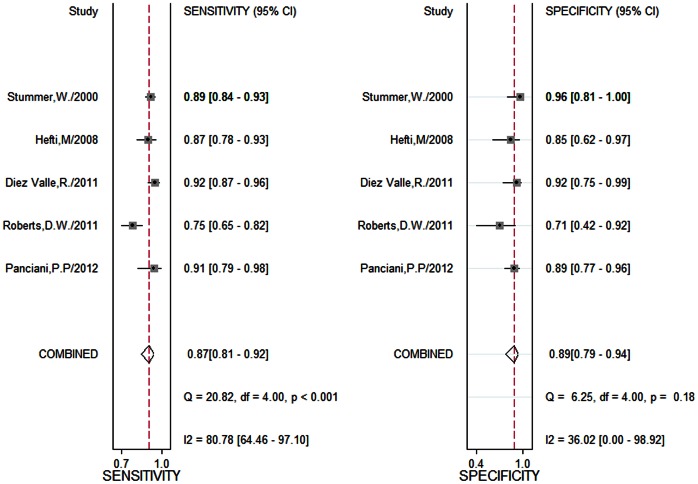
Forest plot for diagnosis of glioblastoma multiforme in studies included in meta-analysis.

**Figure 4:**
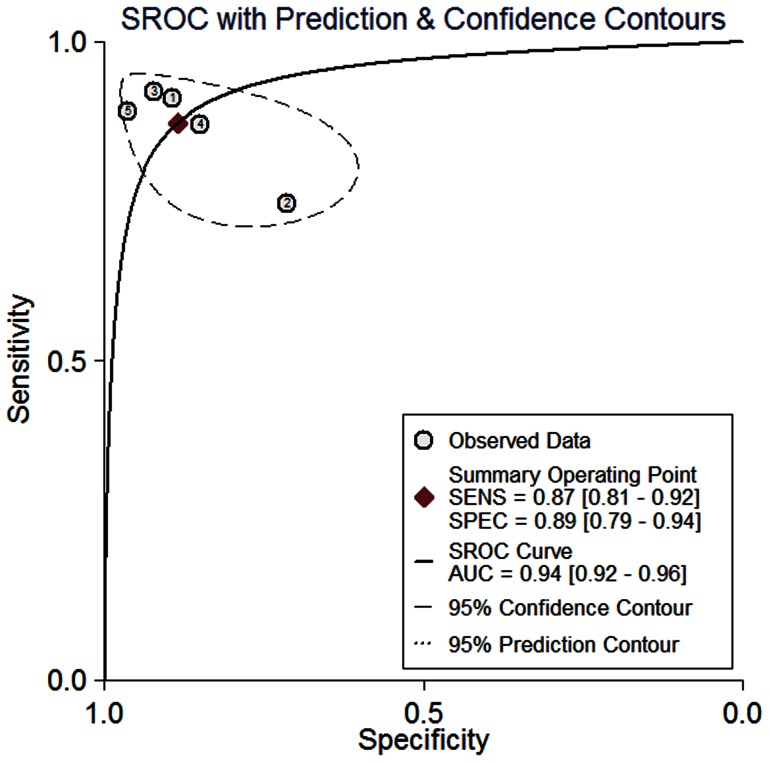
Summary receiver operating characteristic curve for diagnosis of glioblastoma multiforme. Included studies: ①Panciani et al. [19]; ②Roberts et al. [21]; ③Diez Valle et al. [23]; ④Hefti et al. [4]; ⑤Stummer et al. [26].

Figure 3 shows the sensitivity and specificity of included studies, stratified by the reference standard. 5-ALA demonstrated an overall sensitivity of 0.87 (95% CI, 0.81–0.92), specificity of 0.89 (95% CI, 0.79–0.94). A homogeneity test of sensitivity and specificity showed Q = 20.82 (*P*<0.001), *I* ^2^ = 80.78% and Q = 6.25 (*P* = 0.18), *I* ^2^ = 36.02%, respectively. Therefore, notable heterogeneity was detected in the sensitivity analysis. The Positive LR was 7.62 (95% CI, 3.87–15.01), Negative LR was 0.14 (95% CI, 0.09–0.23), and Diagnostic OR was 53.06 (95% CI, 18.70–150.51). The SROC for the diagnosis of GBM versus non brain tumor lesion is presented in Figure 4. The area under the curve of SROC was 0.94.

Two of these studies investigated diagnostic accuracy by examining tissue biopsy samples surgically excised by 5-ALA-induced fluorescence-guided surgery in combination with conventional neuronavigation-guided surgery [4], [19]. These studies demonstrated the ability of 5-ALA to detect more tumor lesions when compared to conventional neuronavigation-guided surgery (Table 4).

**Table 4:** Detection of tumor lesions in comparison of the administration of 5-ALA with conventional neuronavigation.

Publication	5-ALA		Neuronavigation	
	sensitivity	specificity	sensitivity	specificity
Panciani et al. (2012)	91.1%	89.4%	57.8%	57.4%
Hefti et al. (2008)	87.0%	85.0%	66.0%	68.0%

Different fluorescent qualities were observed in GBM patients [4], [21], [23], [26]. Stummer et al. [26] and Hefti et al. [4] showed that viable tumor tissue could be identified by its deep red fluorescence, which they called “solid”, whereas normal brain tissue was colored blue. They also reported a pink fluorescence which was encountered between solidly fluorescing tumor and non-fluorescing blue brain tissue, and they called this kind of fluorescence impression “vague”. Diez et al. [23] also differentiated different fluorescent qualities, with neat distinction of the solid mass of the tumor in bright red, and the border in shades of pink. Roberts et al. [21] assigned biopsy sites a fluorescence level consisting of the following scores: 0, no fluorescence; 1, low fluorescence; 2, moderate fluorescence; or 3, high fluorescence. Two studies calculated sensitivity and specificity according to strong fluorescence and vague fluorescence respectively (Table 5).

**Table 5:** Calculated sensitivity and specificity for strong fluorescence and vague fluorescence.

Publication	Strong fluorescence (bright red)		Vague fluorescence (pink)	
	sensitivity	specificity	sensitivity	specificity
Diez Valle et al. (2011)	85.7%	100.0%	85.4%	92.3%
Hefti et al. (2008)	98.0%	100.0%	76.0%	85.0%

### Assessment of EOTR

Four studies assessed EOTR, which was measured by early postoperative MRI [11], [12], [23], [26]. These studies described how the use of 5-ALA affected EOTR and helped in achieving gross total resection. Three studies were conducted by the ALA-Glioma study group [11], [12], [26]. The study conducted by Stummer et al. in 2011 [12] was a supplemental analysis to a randomised controlled multicentre phase III trial completed by the same group [11].

Stummer et al. [26] prospectively reported that on postoperative MR images, complete resection of contrast-enhancing tumor was observed in 33 (63%) of 52 patients. Areas of residual contrast enhancement were identified by comparing T1-weighted noncontrast-enhanced and contrast-enhanced axial sections (matrix 256×256 pixels, 5-mm slices, 1-mm gap; 0.1 mmol gadolinium-diethylenetriamine pentaacetic acid/kg body weight). Of 17 patients in whom complete removal of fluorescent tissue was achieved, 16 revealed no residual enhancement on postoperative MR images. In the remaining patient, a small region of residual enhancement was found. On the other hand, postoperative MR imaging was also devoid of residual enhancement in nine of 12 patients with vague residual fluorescence but in only eight of 23 patients with solid residual fluorescence.

A randomised controlled multicentre phase III trial conducted by Stummer et al. reported that contrast-enhancing tumor was completely resected in 90 (65%) of 139 patients assigned 5-ALA compared with 47 (36%) of 131 assigned white light (*P*<0.0001) [11]. Stummer et al. subsequently reported that the percentage of patients without residual tumor on early postoperative MR imaging in the ALA group was 63.6%, and it was 37.6% in the WL group (*P*<0.0001) [12]. More frequent complete resections were confirmed, with higher median residual tumor volumes in the WL group (0.5 vs 0 cm^3^, *P* = 0.001). Residual tumor was defined as contrast enhancement with a volume >0.175 cm^3^ in both studies. Diez et al. [23] reported that all the contrast-enhancing volume was resected in 83.3% of the patients, all patients had resection over 98% of the volume and mean volume resected was 99.8%.

### Safety and Survival

Safety regarding neurological function was assessed in four studies by recording AEs, SAEs, the NIH-SS score, and the KPS score [12], [20], [23], [25]. In a multicentric phase II safety study of 5-ALA, 6-week AE incidence in 243 patients evaluable for safety, was 51.9% (nervous system disorders: 30.0%) [20]. Stummer et al. [12] reported that the incidence of AEs (excluding SAEs) was 58.7% in the ALA group and 57.8% in the WL group, as was the incidence in the subgroup of neurological AEs (*P* = 0.74).

Survival data were available in six studies [11], [12], [20], [23], [25], [26], three of which were RCT [11], [12], [23]. Progression-free survival and overall survival in these three studies are shown in Table 6. Patients in 5-ALA group had higher 6-month progression free survival and overall survival than those in white light group.

**Table 6:** Survival data.

Publication	No. of patients	Subgroup	Overall survival (mo)		PFS rate at 6 months	
			5-ALA	WL	5-ALA	WL
Stummer et al. (2011)	349	–	14.3	13.7	46.0%	28.3%
Eljamel et al. (2008)	27	–	12.3	5.6	–	–
Stummer et al. (2006)	270	older	14.1	11.5	41.0%	21.1%
		younger	18.0	17.5		

Abbreviations: Mo, month; PFS, progression-free survival; WL, white light.

## Discussion

To our knowledge, this is the first systematic review to address the (added) value of 5-ALA-guided resection of high-grade malignant gliomas compared with conventional neuronavigation-guided surgery, with respect to diagnostic accuracy, EOTR, safety, and survival. Only trials designed for level 1 and 2 were included. Meta-analysis of data was possible and indicated that 5-ALA-guided surgery can additionally detect residual tumors in patients with high-grade malignant gliomas. Our estimate of the incremental accuracy of 5-ALA, using SROC curves, showed an AUC of 94%. Evidence from these studies also showed 5-ALA-guided surgery enable more complete resections of contrast-enhancing tumor, leading to improved progression-free survival in patients with high-grade malignant glioma. Although 5-ALA allows the neurosurgeon to more accurately distinguish glioma margins intraoperatively, many studies published on 5-ALA-guided resection of glioma have some important limitations.

### Low Sensitivity in Low-grade Gliomas

Different types of gliomas differ substantially in macroscopic and sometimes MRI appearance, and in survival. Our review focused on high-grade gliomas in general, and GBM in particular. Evidence showed that the high specificity and sensitivity of 5-ALA-induced PpIX fluorescence in high-grade gliomas allowed immediate and reliable identification of these tumors. However, visible 5-ALA induced fluorescence is rare in low grade glioma (WHO I and II). Therefore, lower grade glioma cannot be reliably resected by fluorescence-assisted surgery alone. In these cases, the additional use of intraoperatively imaging-based neuronavigational methods (MR, ultrasound) may be required.

### Fluorescent Quality

Many studies reported different qualities of fluorescence induced by 5-ALA were observed in brain tissues, and visible levels of fluorescence correlated with tumor burden and WHO grades [4], [21], [23], [26]. Two types of fluorescence were noted: solid fluorescence and vague fluorescence. Sometimes, the assessment of intraoperative 5-ALA-induced fluorescence by surgeon was largely subjective. This approach is limited in its sensitivity for identifying low levels of fluorescence in tumor, potentially leaving some amount of resectable tumor unidentified [27]. There are two approaches to overcome this limitation. One is quantitative or semi-quantitative measurement of fluorescence intensity during operation, and the other is to enhance PpIX fluorescent quality.

Valdes et al. [27] conducted quantitative ex vivo measurement of PpIX concentration with proliferation index and glioma grades on histopathology, and showed that ex vivo quantitative measurement of PpIX concentration in tissue is more sensitive at identifying both low- and high-grade gliomas than current intraoperative fluorescence imaging. As a result, there remains a critical need to improve intraoperative PpIX fluorescence detection to achieve better sensitivity in determination of brain tumors, subsequently leading to optimal surgical resection. Our group also tried to find an approach to enhance PpIX fluorescent quality for optimising the subjective discrimination of vague fluorescence and improving the effect of 5-ALA [38], [39].

### Assessment of EOTR

A growing body of evidence demonstrated that a more extensive surgical resection could improve outcomes of patients with malignant gliomas [1], [30], [40], [41]. Fluorescence-guided resection could offer real time viewing of the tumor in the operative field, and this should allow neurosurgeon to resect almost all the tumural tissue in most cases. The main approach used to assess EOTR is to examine early postoperative contrast enhancement on T1-weighted MRI. This approach is a rough method that dichotomises gross total resection assessment to yes or no [6]. The MacDonald criteria [42], Response Assessment in Neuro-Oncology criteria (RANO) [43], and Response Evaluation Criteria In Solid Tumors (RECIST) [44] are not useful for postoperative assessment of tumor size because they require spherical tumor. It is clear that contrast enhancement at the border of the resection cavity is not the case [6]. Consequently, more reliable and valid criteria to assess EOTR are necessary for neurosurgical studies.

### Future Research

Based on the results of this systematic review, we have a few recommendations for future studies on 5-ALA-guided resection of gliomas. First, as mentioned previously, widespread use of 5-ALA should be based on evidence. The safety and efficacy of 5-ALA-guided resection of gliomas depend on the better understanding of its underlying mechanisms. Second, the intraoperative fluorescence needs to be quantified and the correlation between fluorescence and its histopathology needs to be further investigated. Third, in this review certain studies could not be used for meta-analysis because some statistical analyses were not available in their studies. In addition, the numbers of trials in meta-analysis were too small to analyze publication bias. A well-designed meta-analysis summarizes the results of multiple studies and can provide valuable information for researchers, policy-makers, and clinicians. However, there are many critical problems in performing them, including poor reporting of results in primary studies, bias and heterogeneity across studies. Especially in prognostic studies, some factors should be considered, including relevant patient details (e.g. age, stage of disease), how the primary endpoint was measured, time of disease recurrence, follow-up time, final disease status, important adjustment factors and treatment received. In this systematic review, for instance, the measurement of EOTR, safety, and survival may differ across studies. No meta-analysis was planned about the assessment of EOTR, safety, and survival, because we did not expect that a valid quantitative data synthesis could be done. There is an obvious need for standardization of the assay procedure and the assessment of the specimens as well as for the initiation of a prospective multi-centre trial to provide definite answers. In this context, higher quality of studies on 5-ALA are necessary to show its added value as compared with standard treatment.

### Conclusion

Based on available literature, there is currently, at best, level 2 evidence that 5-ALA-guided surgery is more effective than conventional neuronavigation-guided surgery in increasing diagnostic accuracy and EOTR, enhancing quality of life, or prolonging survival in patients with high-grade malignant glioma.

## Supporting Information

Appendix S1 **PRISMA Checklist.**
(DOC)
